# Centering Ability and Canal Transportation of Nickel-Titanium (NiTi) Single-File Systems With and Without Glide Path in Extracted Natural Teeth: A Systematic Review and Meta-Analysis

**DOI:** 10.7759/cureus.101131

**Published:** 2026-01-08

**Authors:** Indumathi Manoharan, Deblina Basu, Mathan Rajan

**Affiliations:** 1 Conservative Dentistry and Endodontics, Sri Ramachandra Dental College and Hospital, Sri Ramachandra Institute of Higher Education and Research, Chennai, IND; 2 Conservative Dentistry and Endodontics, Sri Ramachandra Institute of Higher Education and Research, Chennai, IND

**Keywords:** centering ability, extracted natural human teeth, glide path, reciprocating files, rotary file, transportation

## Abstract

Establishing an endodontic glide path is crucial for the preservation of tooth structure and helps to avert various iatrogenic errors, including perforations and instrument fracture. Whether the preparation of the glide path is required before shaping the canals is still debatable. This systematic review aims to assess the centering ability and canal transportation of single-file nickel-titanium (NiTi) instruments with and without glide path in extracted natural teeth. The review was conducted following the Preferred Reporting Items for Systematic Reviews and Meta-Analyses (PRISMA) statement. An electronic database search using PubMed, SCOPUS, EMBASE, Literatura Latino-Americana e do Caribe em Ciências da Saúde (LILACS), Cochrane Library, Google Scholar, and grey literature was performed from inception till August 2025 based on the inclusion and exclusion criteria, and the quality was assessed using the revised Quality Assessment Tool for In Vitro Studies (QUIN) risk of bias tool. Three articles were included for the qualitative synthesis, and two articles were included for the meta-analysis. The results stated that there is less canal transportation and an increased centering ratio in the coronal, middle, and apical third of the canal when glide path preparation was done (p<0.05). The current systematic review provides evidence suggesting that centering ability is significantly higher and canal transportation is significantly lower for NiTi single-file systems when used after glide path preparation as compared to instrumentation without glide path preparation.

## Introduction and background

Maintaining the instruments in the center of the canal is crucial for effective enlargement without compromising the root's structural integrity. Curved root canals pose a risk of canal transportation, especially if clinicians neglect to pre-curve larger-sized instruments. A procedure implemented to decrease this risk was the pre-coronal enlargement of the canal, incorporating scouting, patency, and glide path [1].

Glidepath refers to a seamless radicular pathway that extends from the coronal orifice of a root canal to the apical constriction [2]. The creation of the glidepath is believed to be a crucial stage in root canal instrumentation because it enables all succeeding instruments to travel freely from the coronal orifice of the canal to the apical constriction in an unhindered manner, thereby increasing the longevity of the shaping instruments [3]. A "manual glide path" is prepared with hand files made up of stainless steel. This maintains better tactile sensation, reduces the possibility of instrument fracture, and facilitates easier overcoming of canal obstructions [4].

However, no effect on the degree of dentine removal was found when instrumentation of canals was done with and without a glide path establishment. Many other studies have supported the above result, claiming glide path management (GPM) as a time-consuming endodontic practice that can be overlooked by clinicians due to advancements in nickel-titanium (NiTi) files [5].

The recent advancement in NiTi, such as superior flexibility, is currently developed using proprietary heat treatments and various surface treatments, allowing it to negotiate canal curvatures safely [6]. The advancements in NiTi shaping files eliminate the need for GPM for maintaining the canal centering, but without causing canal transportation, is debatable. Hence, there is a requirement for conducting a systematic review to conclude whether glide path preparation can impact the canal centering ability of the endodontic files and can reduce the canal transportation in curved canals.

## Review

Materials and methods

The protocol for this systematic review has been registered with the PROSPERO International Prospective Register of Systematic Reviews, Registry No. CRD42025638709. A protocol was readied and listed in the Open Science Framework (OSF) database [6,7]. This systematic review was conducted according to the guidelines of the Preferred Reporting Items for Systematic Reviews and Meta-Analyses (PRISMA). This systematic review included in vitro studies comparing centering ability and canal transportation at each third of canals in extracted natural posterior teeth with and without glide path preparation. This systematic review used the Population, Intervention, Comparison, Outcome, and Study (PICOS) framework to develop the research question.

PICO(S) Analysis

The PICO(S) framework was defined as follows: the population (P) comprised extracted natural posterior teeth instrumented using single-file NiTi systems. The intervention (I) was performed with prior glide path preparation, while the comparison (C) group consisted of canals instrumented without glide path preparation. The outcomes (O) assessed were centering ability and canal transportation at the apical, middle, and coronal thirds of the root canals. The study (S) design included in vitro studies.

This systematic review excluded the in vivo studies, in vitro studies utilizing acrylic resin blocks, teeth with immature apex, calcified canals, S-shaped canals, full text not available, case reports and case series, cross-sectional studies and descriptive studies, reviews, laboratory research, animal studies, and studies not in the English language (Table 1).

**Table 1:** Characteristics of excluded studies

S. No.	Author	Year	Reason for exclusion
1	Lim et al. [8]	2012	Resin blocks were used for the study
2	Nazarimoghadam et al. [9]	2014	Resin blocks were used for the study
3	Aydin and Karataslioglu [10]	2016	Resin blocks were used for the study
4	Keskin et al. [11]	2018	Resin blocks were used for the study
5	Tharuni et al. [12]	1996	Resin blocks were used for the study
6	Serene et al. [13]	1995	Study in a language other than English
7	Mais et al. [14]	2024	Clinical study

Methods of Search

Up until August 2025, eligible studies were typically identified through extensive electronic searches of the databases such as PubMed, SCOPUS, EMBASE, Literatura Latino-Americana e do Caribe em Ciências da Saúde (LILACS), Cochrane Library, and Google Scholar. To find new, relevant studies, reference lists from identified in vitro studies and review papers were manually examined. Reading the entire text was also used to evaluate potentially eligible studies, and the final decision regarding addition was made. The search terms in various databases have been listed in the Appendices.

Two reviewers (IM, DB) independently screened the titles and abstracts of all the articles obtained through the initial search. The complete text of relevant studies was assessed. Any disagreement in the selection of studies was resolved with a third reviewer (MR. The following data were extracted from each involved article: year of publication, country where the study was undertaken, study design, natural teeth/resin block used, degree of canal curvature (in degrees), type of NiTi instrument used, type of file used in glide pathway, blinding, centering ability, transportation, and the author’s concluding remarks.

Quality Assessment

Quality Assessment Tool for In Vitro Studies (QUIN) risk of bias tool was utilized to evaluate the risk of bias of the included in vitro studies in this systematic review. The total scores (TS) from the 12 criteria are obtained, and the following formula is used to calculate the final score (FS) percentage: $FS\% = \dfrac{TS \times 100}{2 \times \text{count of applicable criteria}}$. According to this percentage, each study is classified as high, medium, or low when the FS% is >70%, 50%-70%, and <50%, respectively.

Results

Extraction of Data

**Figure 1:**
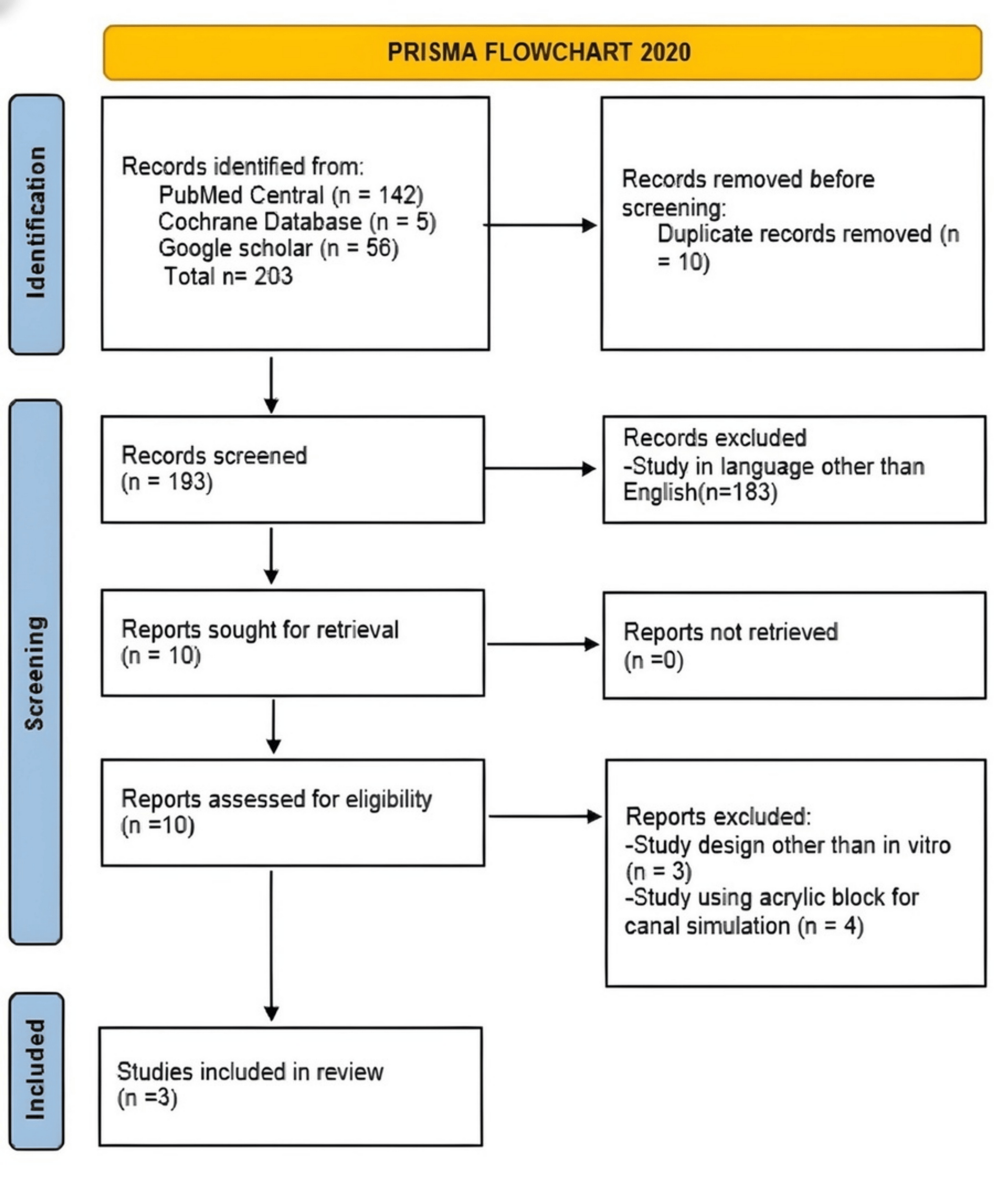
PRISMA flowchart illustrating the study selection process based on the inclusion and exclusion criteria PRISMA: Preferred Reporting Items for Systematic Reviews and Meta-Analyses

The article inclusion procedure is depicted in the PRISMA study flow diagram in Figure 1. Initially, 203 records were screened. The duplicates were removed, and studies were excluded based on title and abstract review. After the process of duplicate removal, 193 articles were identified for screening. Of these, 10 articles were selected for full-text reading as they fulfilled the initial selection criteria with relevance to the title and abstracts. After full-text reading, seven articles were excluded. Three articles that fulfilled the eligibility criteria were finally included for this qualitative review, and two articles were included for the quantitative analysis.

Characteristics of Included Studies

Between 2017 and 2025, three included studies had a total of 240 samples. These samples were roots of maxillary and mandibular molars. The extracted data are tabulated in Table 2.

**Table 2:** Characteristics of the included studies NiTi: nickel-titanium

Author name	Year	Sample size	Natural teeth/resin block used	Degree of canal curvature (in degrees)	Type of NiTi instrument used	Type of file used in the glide pathway	Blinding	Centering ability	Transportation	Conclusion
Sajad et al. [15]	2018	60	Extracted mandibular molars	20-35	F6 Sky Taper File	Path glider size 15 and size 20	Yes	No significant difference	No significant difference	Glide path had no influence on centering ability.
Vorster et al. [16]	2018	60	Extracted mandibular molars	25-35	Primary WaveOne Gold (25/07)	1. K-file size 10, 15, 20; 2. Pathfinder files nos. 1-3; 3. WaveOne Gold Glider no. 1-3.	No	No significant difference in centering ability after four types of instruments are used	Glide path reduces canal transportation	Glide path preparation significantly reduces canal transportation
Hage et al. [17]	2020	120	Extracted maxillary and mandibular molars	25-40	1. Reciproc 25; 2. Reciproc blue 25	Pathfinder files 13/.02, 16/.02, 19/.02	No	Centering ratio was significantly high in glide path preparation groups. Significant difference found in the centering ratio in the four techniques.	Pathfinder files greatly reduced canal transportation	Glide path preparation reduces canal transportation

Intervention

The included in vitro studies compared the centering ability and canal transportation of NiTi instruments used with and without glide path preparation in extracted natural teeth. Glide path preparation was performed using PathGlider files, K-files, Pathfinder files, and WaveOne Gold Glider files. Canal centering ability was evaluated using the Gambill formula, while canal transportation was assessed using photographic analysis, micro-computed tomography (micro-CT), and cone-beam CT (CBCT).

Meta-Analysis

STATA version 17.0 (STATA/SE 17.0 for Windows; StataCorp LLC, College Station, TX, USA) was used for data analysis. Of the three included studies, meta-analysis was performed for two studies due to their methodological homogeneity.

Canal transportation

Coronal Third

**Figure 2:**
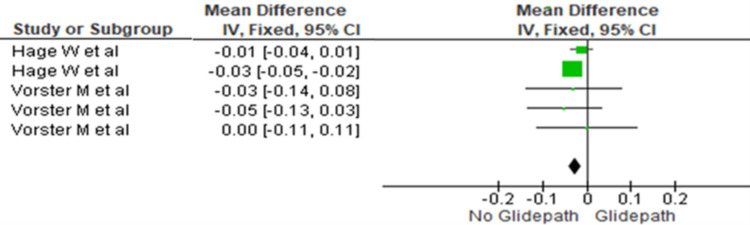
Forest plot depicting canal transportation at the coronal third References [17,16]

The canal transportation was compared between the two groups at the coronal third. The heterogeneity was recorded as 0%; therefore, the fixed effect model was used. The overall mean difference recorded was - 0.03 (-0.04, -0.01). This indicates more canal transportation in the no glide path group. This difference was statistically significant (p<0.05) (Figure 2).

Middle Third

**Figure 3:**
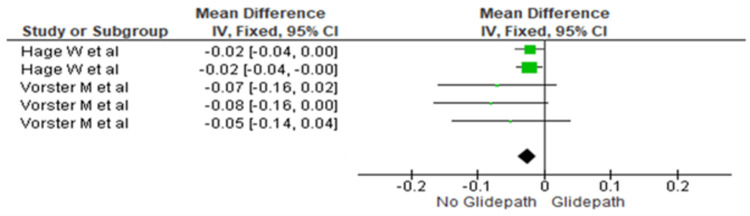
Forest plot depicting canal transportation at the middle third References [17,16]

The canal transportation was compared between the two groups at the middle third. The heterogeneity was recorded as 0%; therefore, the fixed effect model was used. The overall mean difference recorded was -0.02 (-0.04, -0.01). This indicates more canal transportation in the no glide path group. This difference was statistically significant (p=0.0006) (Figure 3).

Apical Third

**Figure 4:**
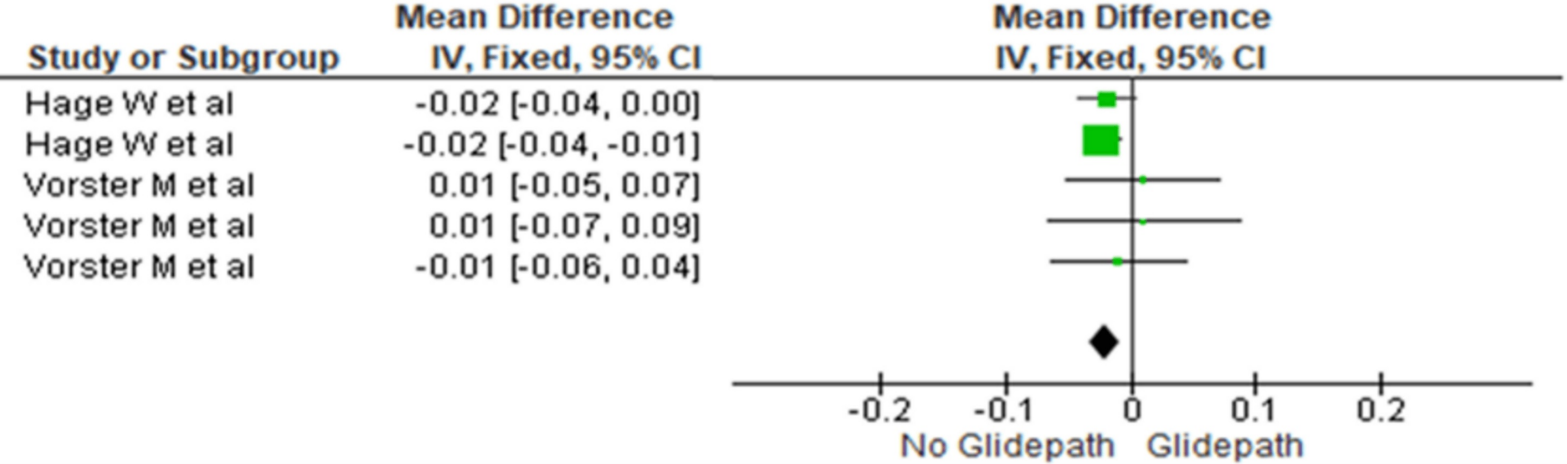
Forest plot depicting canal transportation at the apical third References [17,16]

The canal transportation was compared between the two groups at the apical third. The heterogeneity was recorded as 0%; therefore, the fixed effect model was used. The overall mean difference recorded was -0.02 (-0.03, -0.01). This indicates more canal transportation in the no glide path group. This difference was statistically significant (p=0.0007) (Figure 4).

Canal centering ability

Coronal Third

**Figure 5:**
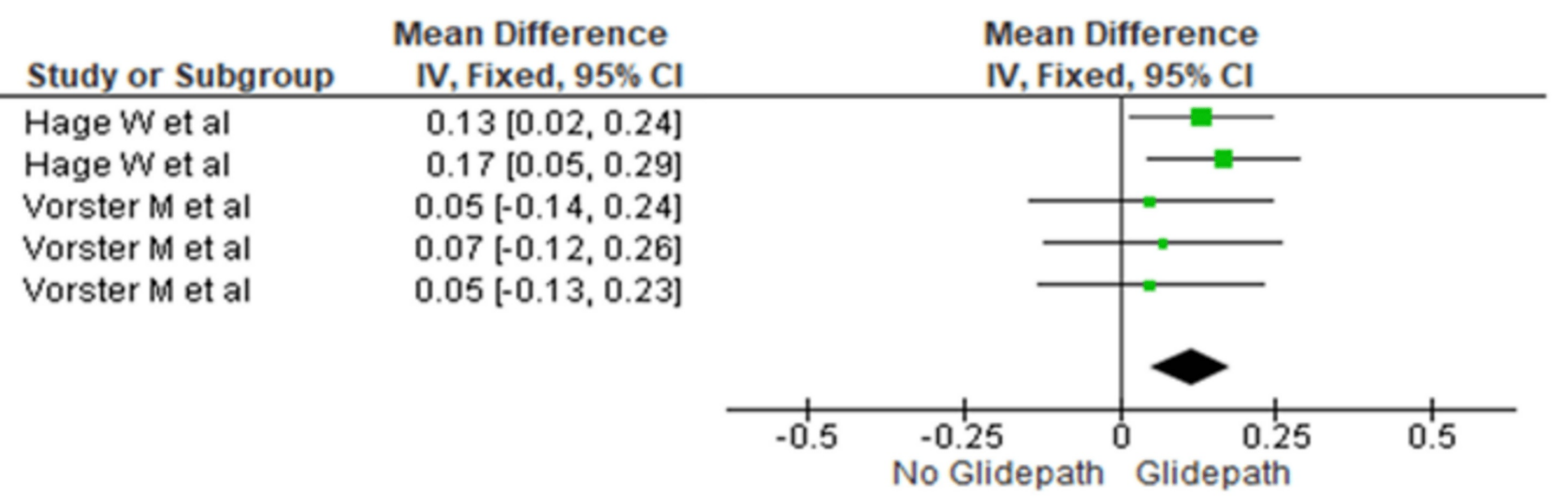
Forest plot depicting canal centering ability at the coronal third References [17,16]

The centering ratio was compared between the two groups at the coronal third. The heterogeneity was recorded as 0%; therefore, the fixed effect model was used. The overall mean difference recorded was 0.011 (0.05, 0.11). This indicates more centering in the glide path group. This difference was statistically significant (p=0.0006) (Figure 5).

Middle Third

**Figure 6:**
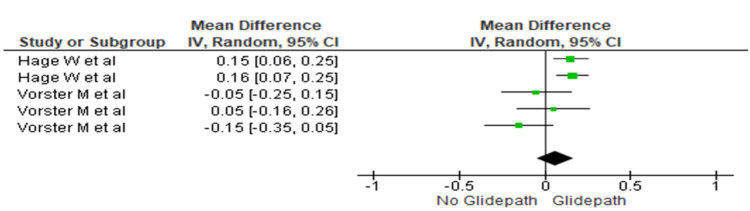
Forest plot depicting canal centering ability at the middle third References [17,16]

The centering ratio was compared between the two groups at the middle third. The heterogeneity was recorded as 65%; therefore, the random effect model was used. The overall mean difference recorded was 0.06 (-0.05, 0.17). This indicates more centering in the glide path group. This difference was statistically significant (p=0.028) (Figure 6).

Apical Third

**Figure 7:**
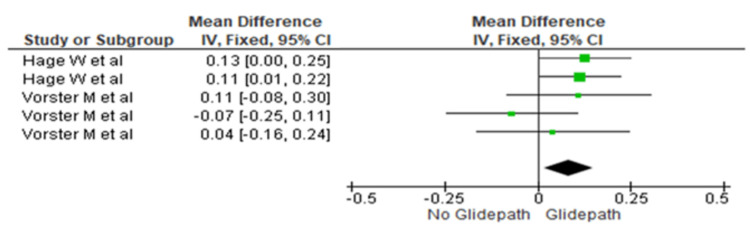
Forest plot depicting canal centering ability at the apical third References [17,16]

The centering ratio was compared between the two groups at the apical third. The heterogeneity was recorded as 0%; therefore, the fixed effect model was used. The overall mean difference recorded was 0.08 (0.02, 0.15). This indicates more centering in the glide path group. This difference was statistically significant (p=0.001) (Figure 7).

The meta-analysis demonstrated significantly greater canal transportation in the no glide path group across the coronal, middle, and apical thirds (Figures 2-4). The mean differences were -0.03 (-0.04 to -0.01) in the coronal third, -0.02 (-0.04 to -0.01) in the middle third, and -0.02 (-0.03 to -0.01) in the apical third, indicating significantly increased transportation without glide path preparation.

In contrast, significantly greater centering ability was observed in the glide path group at all three canal levels (Figures 5-7). The mean differences were 0.011 (0.05, 0.11) in the coronal third (p=0.0006), 0.06 (-0.05, 0.17) in the middle third (p=0.028), and 0.08 (0.02, 0.15) in the apical third (p=0.001).

Outcomes

Out of three studies that compared, it was suggested that in all three, more centering was observed in the glide path group. This difference was statistically significant in the coronal third (p=0.0006), in the middle third (p=0.028), and in the apical third of the canal (p=0.01). Estimates for the risk of bias categories are shown in Table 3. Out of the three studies, two have a moderate risk, and one has a low risk of bias. None of the studies received monetary support or a research grant.

**Table 3:** Risk of bias assessment of the included studies using the QUIN tool QUIN: Quality Assessment Tool for In Vitro Studies

	Sajad et al. [15]	Vorster et al. [16]	Hage et al. [17]
Clearly stated aims/objectives	2	2	2
Sample size calculation	2	2	2
Sampling technique	2	2	2
Details of the comparison group	2	2	2
Detailed explanation of methodology	2	2	2
Operator details	0	0	0
Randomization	2	0	2
Method of measurement of outcome	2	2	2
Outcome assessor details	0	0	0
Blinding	2	0	0
Statistical analysis	2	2	2
Presentation of results	2	2	2
Total score	20	16	18
Final score (%)	83.33%	66.66%	75%

Discussion

NiTi files exhibit shape memory properties that frequently result in the removal of dentin on the outer curve within the apical third of the canal, which causes transportation. According to Wu et al., a deviation greater than 0.3 mm may disrupt the apical seal, eventually leading to microleakage and treatment failure. Therefore, the ability to shape and clean the root canal wall while maintaining the curvature of the canal is considered one of the main parameters used to evaluate a NiTi root canal instrumentation file, increasing success rates [18].

Since there are two different research outcomes regarding GPM, the present systematic review assesses the centering ability and canal transportation of NiTi instruments with and without glide path in extracted natural teeth.

Included in this systematic review are three in vitro studies that used a single-file NiTi system after a glide path file. The QUIN tool has been used in this systematic review for risk of bias quality assessment [19]. Among the three studies included, two had a low risk of bias, and one study had a medium risk of bias. In our systematic review, two studies have used reciprocating, whereas one study utilized a rotary file after creating a glide path for canal preparation. Due to homogeneity, two studies were subjected to meta-analysis. The results suggested that the centering ability of NiTi instruments resulted in significantly more centering and reduced canal transportation in the glide path group at the coronal, middle third, and apical third of the canal, which was statistically significant (p<0.05).

Hage et al. and Vorster et al. studies used PathFile for GPM. PathFile rotary system (Dentsply, Sirona, Bensheim, Germany) includes three files of 0.02 constant taper (#13.02, #16.02, #19.02) with a square cross sec­tion and a guiding tip design reducing the risk of ledges and canal transportation [20]. Employing this glide path NiTi system before the application of a single shaping file improves the adherence to the original anatomical structure [21]. Sajad et al. used the PathGlider file system for GPM, having a non-cutting tip manufactured using proprietary heat-treatment. It features a square cross-sectional shape with a tip diameter of 0.16 mm and a progressive taper (0.02 at the tip level increasing to 0.085), designed to enlarge the coronal and middle sections of the root [22]. Stainless Steel files are rigid, whereas NiTi rotary files (PathFile and PathGlider) offer superior flexibility and low modulus of elasticity, which preserves dentin. PathGlider is generally considered superior to PathFile because its single-file system, progressive taper, and use of M-Wire technology offer faster preparation time, better cyclic fatigue resistance, and demonstrated diminished canal transportation compared to the three-file, constant-taper PathFile system [23].

The glide path preparation using glide path files of a lesser core diameter and increased flexibility helps in establishing a definitive pathway with lesser deviation from the original canal anatomy. This pathway, when followed by instrumentation using greater taper files, compels the files to follow the previously created pathway, which provides the pathway of least resistance, reducing the chance of deviation from original canal anatomy [24].

Apart from glide path preparation, several factors significantly influence the likelihood of canal transportation. These include the root canal anatomy, specifically the degree and radius of curvature, where a smaller radius indicates a higher risk for canal straightening after instrumentation [25]. The design of the file is also critical, encompassing features like the tip, cross-sectional design, and taper; files with a non-cutting tip are preferred as they cause less transportation and promote more uniform dentin removal in the canal. Finally, the NiTi alloy itself is a factor, as its properties are defined by its microstructural phase (rigid austenite versus flexible martensite). Specific heat treatments are used to modify the alloy's composition, tailoring the instrument's flexibility to ensure it remains centered during rotation, thereby minimizing alterations to the canal's original anatomy [26].

Coronal preflaring has been done in previous studies. It has been studied that coronal preflaring prior to glide path preparation leads to a significant loss of pericervical dentin and, in turn, decreases the fracture resistance of an endodontically treated tooth. For biomechanical preparation, sequential files have been used (RaceEVO and Edge Sequel Sapphire) [27,28]. In the present review, we have included articles using a single NiTi file for biomechanical preparation.

This review is distinct from earlier reviews because it evaluates canal centering and transportation utilizing acrylic endodontic training blocks (AETB) and S-shaped canals, which are excluded from our review [29]. Furthermore, other reviews encompassed studies that compared canal centering and transportation with canal curvatures ranging from 25° to 76° [30]. In contrast, the current review focuses on studies with curvatures between 20° and 40°. The studies included have factored in preflaring before GPM [31], which was not a criterion for the present systematic review.

Within the limitations of this systematic review, the establishment of a reproducible glide path is considered a critical prerequisite for the safe and effective shaping of root canals. It is associated with the mitigation of procedural errors, enhancing the long-term prognosis and overall success rate of endodontic treatment.

## Conclusions

This systematic review currently provides evidence that suggests a significant enhancement in centering ability and a notable reduction in canal transportation of single-file NiTi instruments in the coronal, middle, and apical thirds of the root canal when glide path preparation is utilized, as opposed to when it is not. Furthermore, the single-file system is generally regarded as superior to the sequential file system as it offers better resistance to cyclic fatigue and demonstrates lower canal transportation.

## Appendices

**Table 4:** Search terms used for the various databases

Database	Search terms
PubMed	((((natural teeth)OR(simulated canals))OR (extracted teeth)OR(curved canals))AND( with glide path)OR(without glide path)AND(centering ability) OR(canal transportation))AND( in vitro studies)
Cochrane Library	natural teeth or extracted teeth or with glide path or without glide path or canal transportation or centering ability
Google Scholar	Natural teeth + extracted teeth + simulated canals + with glide path + without glide path + in vitro studies + reciprocating instruments + centering ability + canal transportation + ex vivo studies + canal centering + reciproc + waveone
